# COVID-19 reveals Brugada pattern in an adolescent patient

**DOI:** 10.1017/S1047951120002619

**Published:** 2020-08-03

**Authors:** Nak Hyun Choi, Eric S. Silver, Michael Fremed, Leonardo Liberman

**Affiliations:** Division of Paediatric Cardiology, Morgan Stanley Children’s Hospital of New York-Presbyterian, Columbia University Medical Center, New York, New York, USA

**Keywords:** Brugada, coronavirus, electrocardiogram

## Abstract

A diagnosis of Brugada pattern in paediatric or adolescent patients is rare. COVID-19 is characterised by fevers and a pro-inflammatory state, which may serve as inciting factors for Brugada pattern. Recently described in two adult patients, we report the first case of Brugada pattern in an adolescent with COVID-19.

## Case report

A 19-year-old Hispanic male presented to the paediatric emergency room with 7 days of fever and a daily maximum temperature of 102°F (38.9°C). Over the 72 hours prior to presentation, he developed bilateral shoulder pain followed by progressively worsening chest pain, cough, and shortness of breath.

In the emergency room, he was afebrile but noted to be tachycardic (117 beats/minute), hypertensive (140/91 mmHg), and hypoxaemic (SpO_2_ 94%). On examination, he had increased work of breathing and diminished aeration in the basilar lung fields bilaterally.

His past medical history was notable only for obesity (body mass index > 30 kg/m^2^) and obstructive sleep apnoea without a history of syncope or seizures. He was born from a consanguineous union between parents of South American descent. Family history included a maternal uncle who had epilepsy during childhood and a paternal grandfather who died at an early age with unknown aetiology.

**Figure 1.**
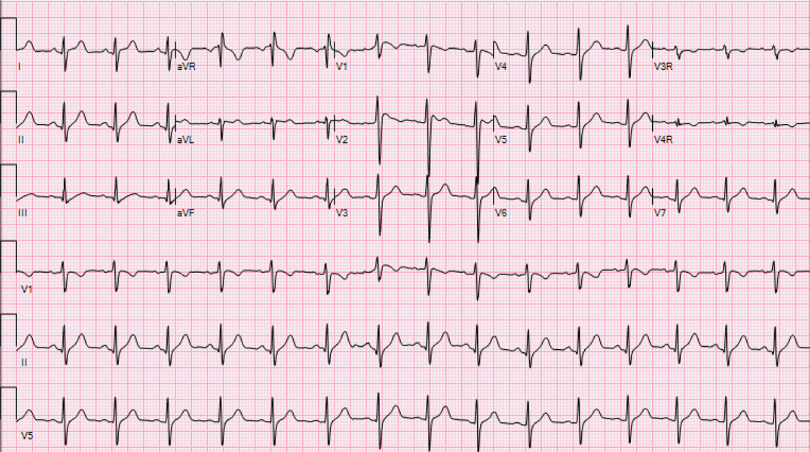
Initial electrocardiogram demonstrating absence of Brugada type 1 pattern.

His initial work up revealed mild elevation of transaminases, mildly elevated ferritin level (415.6 ng/mL), and a chest X-ray demonstrating ill-defined bilateral opacities. Initial electrocardiogram in the emergency room showed normal sinus rhythm, rightward axis, and QTc interval of 446 ms (Fig 1). His high-sensitivity troponin level was within the normal range along with normal inflammatory markers and electrolytes. The patient tested positive for severe acute respiratory syndrome coronavirus 2 by reverse transcriptase polymerase chain reaction.

The patient was admitted to the hospital for monitoring and treatment of his respiratory status. Within 8 hours of hospitalisation, the patient rapidly decompensated, developing persistent hypoxaemia, which ultimately necessitated intubation. Over the next several days, he had persistently high fevers with a maximum temperature of 103.6°F (39.7°C). Following early COVID-19 management guidelines for hospitalised patients with pneumonia, he received hydroxychloroquine and underwent serial electrocardiograms to monitor his QTc duration during treatment.^1^

**Figure 2.**
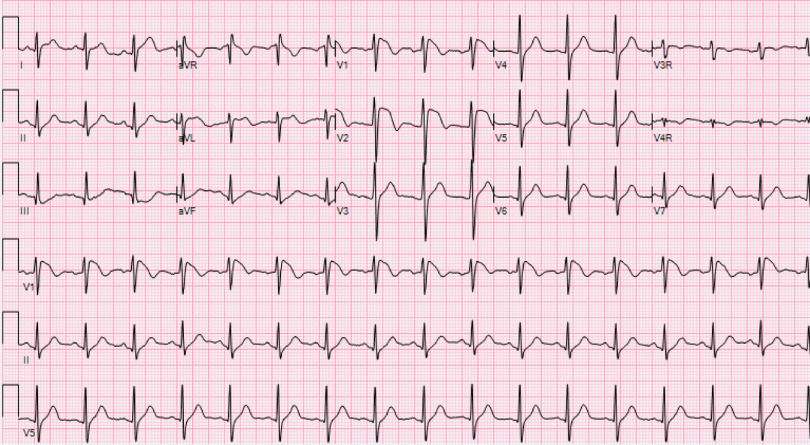
Electrocardiogram demonstrating Brugada type 1 pattern.

On day 3 of hospitalisation, his electrocardiogram showed new ST-elevations (>2 mm) in leads V1 and V2 with a negative T-wave consistent with type 1 Brugada pattern (Fig 2). His laboratory values showed increased inflammatory markers with C-reactive protein of greater than 300 mg/L (normal range: 0–10 mg/L), ferritin of 695 ng/mL (normal range: 30–400 ng/mL), interleukin-6 of 163 pg/mL (normal range <5 pg/mL), and procalcitonin of 0.85 (normal range < 0.08 ng/mL). His electrolytes were normal at the time of the first abnormal electrocardiogram. Followed with serial electrocardiograms, his Brugada pattern initially resolved by day 10 of hospitalisation along with improved inflammatory markers (C-reactive protein of 88.8 mg/L and interleukin-6 of 16.8 pg/mL). On day 18 of his hospitalisation, he had recurrent fever with maximum temperature of 105.6°F (38.9°C) with worsening inflammatory markers (C-reactive protein > 300 mg/L) and recurrence of Brugada pattern on electrocardiogram. His clinical status slowly improved without subsequent Brugada pattern changes, and he was able to be discharged from the hospital.

During his hospitalisation, patient’s genetic testing of the 17-gene Brugada syndrome panel showed a variant of uncertain significance in the SCN5A gene (c.4916G>A; p.G1639E).

## Discussion

First described in 1992, Brugada syndrome is an inherited arrhythmia syndrome, transmitted in an autosomal dominant manner.^2^ Despite its hereditary nature, most patients are not diagnosed until middle adulthood after presenting with life-threatening arrhythmias, including polymorphic ventricular tachycardia or ventricular fibrillation.^3^ The hallmark electrocardiographic finding of the Brugada pattern is ST-segment elevation of the right pre-cordial leads (V1 and V2) that presents spontaneously or is provoked with Class I antiarrhythmic medications.^4^ In children and adolescents, most patients are diagnosed with a surveillance electrocardiogram obtained in the context of a known family history of Brugada syndrome.^5^ Without a family history, the diagnosis can be challenging as the Brugada pattern can be concealed in the absence of a trigger, such as fever.^5^

Mediated by a cytokine-induced inflammatory cascade, COVID-19 incites high fevers, potentially instigating Brugada pattern changes. This change may be secondary to the temperature-dependent, pre-mature sodium channel inactivation found in SCN5A mutation, which is a major ionic abnormality responsible for the phenotype of Brugada pattern.^6^ Recent publications by Chang et al. followed by Vidovich describe two adult patients, aged 49 and 61 years, with Brugada pattern in setting of COVID-19 illness.^7,8^ While any disease causing fever can unmask a Brugada pattern aside from COVID-19 infection, a higher incidence of Brugada pattern may be seen due to the recurrent, high-inflammatory state in patients with COVID-19, along with frequent electrocardiogram monitoring given previously documented cardiovascular complications during acute illness.^9^

New clinical manifestations continue to arise for COVID-19 including recently described multisystem inflammatory syndrome in Children, a manifestation of severe acute respiratory syndrome coronavirus 2, mimicking Kawasaki disease.^10^ In the era of limited, but evolving understanding of this viral illness, clinicians should have a high suspicion for new clinical manifestations in setting of COVID-19. This case highlights the critical importance of assessing for diseases that may be incited by heightened inflammatory states.

Additionally, prompt diagnosis of Brugada pattern in COVID-19-positive patients can have important clinical impact. In our case, we advised the primary intensive care team to avoid certain medications such as Propofol, commonly used for sedation, and Bupivacaine, often used for local anaesthetic, which are typically avoided in Brugada syndrome due to the increased risk of arrhythmia. In the context of the initial reports of Brugada pattern in COVID-19 patients, clinicians should provide close monitoring and direct counselling for patients with a personal or family history of Brugada syndrome or family history of sudden death.
